# A multicenter multistate modeling on the natural history of liver decompensation: Longitudinal analysis of 1777 patients

**DOI:** 10.1097/HC9.0000000000001042

**Published:** 2026-09-28

**Authors:** Eunice X. X. Tan, Nicole Shu Ying Tang, Nicholas Syn, Daniel Q. Huang, Anand V. Kulkarni, Ming-Hua Zheng, Vincent L. Chen, Chitchai Rattananukrom, Jimmy Che-To Lai, Karn Wijarnpreecha, Rahul Kumar, Apichat Kaewdech, Masayuki Ueno, Akash Roy, Ken Liu, Toru Nakamura, Yanhang Gao, Masato Yoneda, Yuya Seko, Takefumi Kimura, Mazen Nourredin, Mohammad Shadab Siddiqui, Hirokazu Takahashi, Cheng Han Ng, Mark Muthiah, the CORE Consortium, Margaret L. P. Teng

**Affiliations:** 1 National University Centre for Digestive Health, National University Hospital, Singapore, Singapore; 2 Yong Loo Lin School of Medicine, National University of Singapore, Singapore, Singapore; 3 National University Centre for Organ Transplantation, National University Health System, Singapore, Singapore; 4 Department of Gastroenterology and Hepatology, Yokohama City University School of Medicine, Kanazawa-ku, Yokohama, Japan; 5 Department of Hepatology and Liver Transplant, Asian Institute of Gastroenterology, Hyderabad, India; 6 MAFLD Research Center, Department of Hepatology, The First Affiliated Hospital of Wenzhou Medical University, Wenzhou, China; 7 Key Laboratory of Diagnosis and Treatment for The Development of Chronic Liver Disease in Zhejiang Province, Wenzhou, China; 8 Division of Gastroenterology and Hepatology, Department of Internal Medicine, University of Michigan Medical School, Ann Arbor, Michigan, USA; 9 Division of Gastroenterology and Hepatology, Department of Medicine, Faculty of Medicine, Srinagarind Hospital, Khon Kaen University, Khon Kaen, Thailand; 10 Medical Data Analytics Centre, Department of Medicine and Therapeutics, The Chinese University of Hong Kong, Hong Kong, China; 11 Division of Gastroenterology and Hepatology, Department of Medicine, University of Arizona College of Medicine, Phoenix, Arizona, USA; 12 Department of Internal Medicine, Banner University Medical Center, Phoenix, Arizona, USA; 13 Department of Transplant, Mayo Clinic, Jacksonville, Florida, USA; 14 Department of Gastroenterology, Changi General Hospital, Singapore, Singapore; 15 Gastroenterology and Hepatology Unit, Division of Internal Medicine, Faculty of Medicine, Prince of Songkla University, Songkhla, Thailand; 16 Department of Gastroenterology and Hepatology, Graduate School of Medicine, Kyoto University, Kyoto, Japan; 17 Department of Gastroenterology and Hepatology, Kurashiki Central Hospital, Kurashiki, Japan; 18 Institute of Gastrosciences and Liver Transplantation, Apollo Multispeciality Hospitals, Kolkata, India; 19 A.W. Morrow Gastroenterology and Liver Centre, Australian Liver Transplant Unit, Royal Prince Alfred Hospital, Sydney, New South Wales, Australia; 20 Sydney Medical School, University of Sydney, Sydney, New South Wales, Australia; 21 Division of Gastroenterology, Department of Medicine, Kurume University School of Medicine, Kurume, Japan; 22 Department of Hepatology, The First Hospital of Jilin University, Jilin University, Changchun, Jilin, China; 23 Molecular Gastroenterology and Hepatology, Graduate School of Medical Science, Kyoto Prefectural University of Medicine, Kyoto, Japan; 24 Department of Medicine, Division of Gastroenterology and Hepatology, Shinshu University School of Medicine, Matsumoto, Japan; 25 Department of Medicine, Sherrie and Alan Conover Center for Liver Disease and Transplantation, Houston Methodist Hospital, Houston, Texas, USA; 26 Houston Research Institute, Houston, Texas, USA; 27 Division of Gastroenterology and Hepatology, School of Medicine, University of Virginia, Charlottesville, Virginia, USA; 28 Liver Center, Saga University Hospital, Saga, Japan; 29 Division of Metabolism and Endocrinology, Department of Medicine, Faculty of Medicine, Saga University, Saga, Japan

**Keywords:** alcohol-associated liver disease, chronic liver disease, decompensation, liver failure, metabolic dysfunction–associated steatotic liver disease, viral hepatitis

## Abstract

**Background:**

Cirrhosis often remains silent until decompensation, after which survival deteriorates sharply. Contemporary, transition-aware estimates are limited, especially in Asian-predominant cohorts. We analyzed trajectories from compensated advanced chronic liver disease (cACLD) through sequential decompensations to death.

**Method:**

In this multicenter study, we analyzed retrospective data from an adult cohort with liver stiffness ≥15 kPa from vibration-controlled transient elastography across 19 institutions internationally. Decompensation included ascites, hepatic encephalopathy, spontaneous bacterial peritonitis, or variceal bleeding. Multistate models were constructed to characterize patient trajectories across 4 main clinically meaningful states: baseline cACLD, first decompensation, a subsequent second decompensation, and death. Etiology-specific subgroup analyses were conducted, and the effects of univariable predictors on transitions were also examined.

**Results:**

Among 1777 patients, 265 (14.9%) developed a first decompensation over a median follow-up of 4.25 years. In the main multistate analysis cohort, from baseline cACLD, first decompensation occurred in 215 patients at 3.10 per 100 person-years, while death occurred at a rate of 1.01 per 100 person-years. The rate of a second decompensation was higher following the first decompensation, at 42.73 per 100 person-years. Patients with alcohol-associated liver disease had the highest rate of progression from baseline to first decompensation, whereas patients with hepatitis B etiology had the lowest progression rate into a second decompensation following a first decompensating event.

**Conclusion:**

Most patients with cACLD remain compensated over 10 years of follow-up, but once decompensation occurs, trajectories accelerate, and survival worsens. Transition-aware, etiology-stratified estimates enable individualized prognostication and earlier risk-guided intervention, including timely transplant referral.

## INTRODUCTION

Liver cirrhosis is frequently unrecognized until clinical decompensation, indicated by cirrhosis-related complications, including ascites, hepatic encephalopathy (HE), spontaneous bacterial peritonitis, and/or variceal bleeding.^1^^–^^3^ Although only a minority of patients die following the first decompensating event, this transition from compensated cirrhosis to decompensated cirrhosis also heralds accelerated morbidity and mortality.^4^ Prognosis is favorable during compensated cirrhosis, with a median survival of more than 12 years and a low 3-year risk of decompensation. In contrast, patients with decompensated liver disease have a median survival as low as 2 years.^2^^,^^5^^,^^6^ The natural history of patients with cirrhosis has been previously well described; however, changing epidemiology and therapeutic advances in recent years justify its re-evaluation.^3^^,^^7^

Moreover, Asian representation in these cohorts is limited, despite the regional burden of liver disease.^8^^–^^10^ Multistate and competing-risk frameworks have been advocated to best map the understanding of this complex disease trajectory in a granular manner, to provide accurate estimates of transition-specific hazards, inter-event times, and the probability of occupying each state over time.^11^^,^^12^ Yet, most epidemiological studies studying cirrhosis reported time-to-first event, and multistate frameworks recommended for this sequential disease were used less frequently.^11^ The primary aim of this study was to delineate the natural history of patients with compensated advanced chronic liver disease (cACLD), through developing a multistate model, characterizing patient trajectories across clinically meaningful states: baseline cACLD, first decompensation, subsequent second decompensation, and death. We further examined variation in disease trajectories by liver disease etiology and evaluated patient-level factors associated with transition-specific progression. We also estimated 1-year, 3-year, and 5-year state occupancy probabilities to provide clinically interpretable estimates of disease course over time.

## METHODS

### Study design

In this retrospective multicenter longitudinal cohort study, we included consecutive adult participants aged 18 years and above with chronic liver disease. Participants were included in the study if they were found to have a vibration-controlled transient elastography (VCTE)–derived liver stiffness measurement (LSM) of 15 kPa or above, at 19 healthcare institutions across North America, Australia, and Asia: Australia (Royal Prince Alfred Hospital, University of Sydney); China (Jilin University, Wenzhou Medical University); Hong Kong (Chinese University of Hong Kong); Japan (Kurashiki Central Hospital, Kurume University, Kyoto Prefectural University, Saga University, Shinshu University, Yokohama City University); India [AIG Hospitals, Apollo Hospitals (Kolkata)]; Singapore (Changi General Hospital, National University Hospital); Thailand (Khon Kaen University, Prince of Songkla University); and the United States of America (University of Michigan, University of Arizona) (Supplemental Table S1, https://links.lww.com/HC9/C482). Clinical, laboratory, and imaging data were collected through review of medical records under a standardized case reporting form and definitions. This study was approved by each center’s institutional review board and was performed as per applicable regulatory requirements and the ethical principles of the Declaration of Helsinki. De-identified data were transferred to and analyzed at the National University of Singapore.

### Study objectives

The primary objective of this study was to delineate the natural history of patients with cACLD, through developing a multistate model characterizing patient trajectories across 4 main clinically meaningful states: baseline (compensated disease), a first decompensation, a subsequent second decompensation, and death—involving a total of 5 allowed transitions: (1) from baseline cACLD to first decompensation, (2) from baseline cACLD to death, (3) from first decompensation to second decompensation, (4) from first decompensation to death, and (5) from second decompensation to death. Secondary objectives included comparing trajectories across etiologies of liver disease to explore heterogeneity across etiologies, as well as examining predictor effects on transitions to identify potential associations.

### Study population criteria and definitions

Consecutive adult patients who presented to any of the participating institutions and had VCTE recorded between 12th May 2008 and 24th April 2025 were included. Patients were not restricted to newly diagnosed cases, and some may have been under clinical follow-up before the index VCTE assessment. Patients in this study underwent VCTE based on clinician judgment and local practice. Patients were excluded from the analysis if they fell under any of the following criteria: (1) age under 18 years at presentation, (2) LSM <15 kPa, (3) missing data on relevant timepoints or dates, and/or (4) had experienced decompensation events at or before baseline. In the main multistate model, patients were additionally excluded if they had experienced eventual hepatocellular carcinoma (HCC) development or underwent eventual liver transplantation during follow-up, as such events were relatively sparse for modeling as additional states. However, such patients were retained for a sensitivity analysis incorporating HCC development and liver transplantation as absorbing states. A decompensation event was defined as the incidence of any (or multiple) of the following clinical events: variceal bleeding, ascites [with or without spontaneous bacterial peritonitis (SBP)], and/or HE.^5^^,^^6^ Body mass index (BMI) was classified into underweight (<18.5 kg/m^2^), normal (18.5–25.0 kg/m^2^), overweight (25.0-30.0 kg/m^2^), and obese (≥30.0 kg/m^2^) for non-Asian patients; cutoffs of 18.5, 23.0, and ≥27.5 kg/m^2^ for each respective category were used for Asian patients. Etiological classifications included metabolic dysfunction–associated steatotic liver disease (MASLD), hepatitis B virus (HBV), hepatitis C virus (HCV), and alcohol-associated liver disease (ALD). MASLD was defined as evidence of hepatic steatosis with exclusion of significant alcohol consumption (≥14 drinks/week for men or ≥7 drinks/week for women) as well as other alternative causes of hepatic steatosis, such as viral hepatitis.^13^ In addition, patients with type 2 diabetes mellitus (T2DM) or obese BMI that had previously been classified under cryptogenic etiology were reclassified as MASLD etiology, based on recommendations from a multi-stakeholder forum.^14^^,^^15^ Non-selective beta blockers (NSBBs) are composed of carvedilol and propranolol. At each participating center, the treatment of cirrhosis was based on the standard of care according to the available recommendations at the time of follow-up. The index date was defined as the date of the VCTE performed at baseline.

### Statistical analysis

All statistical analyses were conducted in R version 4.4.3. Baseline characteristics of the study population were calculated, and continuous variables were presented with median values and interquartile range (IQR). Multistate models with a clock-reset time scale (ie, time since entry into the current state) and adjusting for age at index and sex were fitted for the overall cohort using the *mstate* package, as well as in restricted subsets of the cohort, each containing individuals with only a single specific etiology (MASLD, HBV, HCV, or alcohol-associated). From the fitted models, we estimated the state occupancy probability at various prespecified timepoints. State occupancy probabilities represent the probability of being in a given state at a specific time point, rather than the cumulative probability of ever having experienced a transition into that state. Separately, cumulative incidence of first and second decompensation was estimated using a competing-risk framework, with death before decompensation treated as a competing event. Cumulative incidence estimates represent the probability of having experienced the specified decompensation event by a given time point, regardless of whether the patient subsequently transitioned to another state. Univariable models were fitted to estimate the hazard ratio (HR) for the association of each individual predictor and each transition. Age, BMI, Model for End-Stage Liver Disease (MELD) score, international normalized ratio (INR), platelet count, creatinine, sodium, bilirubin, albumin, and liver enzymes were modeled as continuous variables; other predictors were modeled as categorical variables. In each analysis, individuals were excluded from the corresponding study cohort if data were unavailable for any of the specific variables involved in the model. Point estimates with 95% confidence intervals were reported, and 0.05 was taken as the *p*-value threshold for statistical significance. Stacked probability plots were generated to illustrate state occupancy probabilities over time, representing the estimated probability of being in each state at a given time point, conditional on the specified starting state or time origin. To assess the potential impact of excluding patients who developed HCC or underwent liver transplantation during follow-up, we performed a sensitivity analysis incorporating these outcomes into the multistate framework. In this expanded model, HCC and liver transplantation were included as additional absorbing states, alongside death.

## RESULTS

### Characteristics of the study cohort

A total of 1777 patients from 18 institutions (Supplemental Table S1, https://links.lww.com/HC9/C482) were included in the final cohort for this study. One institution was not represented in the final cohort as none of its enrolled subjects fulfilled the inclusion/exclusion criteria. The median age was 58 (IQR: 50–65) years, and almost half (48%) were obese. The median BMI was 27.3 kg/m^2^. In all, 57% of the patients were males, and 64% of patients had diabetes mellitus at baseline. Two-fifths (41%) of the patients had MASLD alone as the etiology of liver disease, followed by multiple etiologies (23%) and HCV alone (11%). When stratified by time period, the distribution of liver disease etiologies differed. For example, 14.3% of patients included before 2015 had HBV-only etiology, compared with only 7.7% in those included 2015 and after (Supplemental Figure S1, https://links.lww.com/HC9/C482). At the time of inclusion into the study, one-quarter (27%) of patients were prescribed NSBB, and one-third (37%) had endoscopic evidence of esophageal or gastric varices (Table 1). Two-fifths (40.7%) of patients had LSM >25 kPa, and only 31.4% of those who had LSM >25 kPa were prescribed NSBBs. The median follow-up was 4.25 (IQR: 1.47–7.04) years. Of those with first decompensating events, the most common event was ascites with or without SBP (57.0%), followed by variceal bleeding (24.2%), then HE (21.9%). For second decompensating events, the prevalence was 60.2%, 22.3%, and 22.3% for ascites with or without SBP, HE, and variceal bleeding, respectively.

**TABLE 1:** Baseline characteristics of the cohort at the index as stratified by decompensation event(s)

Characteristic	Overall (n=1777)	No decompensation (n=1512)	≥1 Decompensation event (n=265)	*p* ^a^
Age, y^b^	58.0 (50.0, 65.1)	58.0 (50.0, 66.0)	56.0 (49.0, 64.0)	0.151
BMI, kg/m^2^ ^b^	27.3 (24.1, 31.8)	27.6 (24.3, 32.1)	25.7 (23.0, 29.4)	**<0.001**
Sex (female), n (%)	768 (43%)	639 (42%)	129 (49%)	0.053
Ethnicity (Asian), n (%)	1646 (93%)	1399 (93%)	247 (93%)	0.696
Diabetes, n (%)	1136 (64%)	976 (65%)	160 (60%)	0.192
Hypertension, n (%)	1032 (58%)	913 (60%)	119 (45%)	**<0.001**
Hyperlipidemia, n (%)	1004 (56%)	892 (59%)	112 (42%)	**<0.001**
Obesity, n (%)	763 (48%)	676 (50%)	87 (35%)	**<0.001**
LSM (kPa)^b^	22.4 (17.9, 32.0)	21.8 (17.6, 29.5)	27.7 (21.6, 38.9)	**<0.001**
MELD score^b^	8.0 (7.0, 11.0)	8.0 (7.0, 11.0)	10.0 (8.0, 13.0)	**<0.001**
INR^b^	1.1 (1.0, 1.2)	1.1 (1.0, 1.1)	1.2 (1.1, 1.3)	**<0.001**
Platelet (×10^9^/L)^b^	152.0 (102.0, 208.0)	159.5 (112.0, 218.8)	107.0 (76.5, 153.0)	**<0.001**
Creatinine (μmol/L)^b^	67.0 (52.2, 84.0)	67.0 (52.4, 83.1)	69.0 (52.0, 88.4)	0.511
Sodium (mmol/L)^b^	139.0 (137.0, 141.0)	139.0 (137.0, 141.0)	139.0 (136.0, 141.0)	**<0.001**
Bilirubin (μmol/dL)^b^	18.0 (12.0, 30.0)	17.1 (12.0, 27.8)	24.0 (14.0, 39.3)	**<0.001**
Albumin (g/L)^b^	41.0 (38.0, 44.0)	42.0 (38.0, 44.0)	38.0 (34.0, 42.0)	**<0.001**
ALT (U/L)^b^	48.0 (29.0, 84.0)	48.0 (29.0, 85.0)	45.0 (27.0, 75.2)	0.185
AST (U/L)^b^	51.0 (35.0, 81.0)	50.0 (34.0, 80.0)	55.0 (39.0, 90.5)	**0.001**
ALP (U/L)^b^	101.0 (77.0, 140.0)	97.0 (75.0, 134.4)	123.0 (94.0, 167.0)	**<0.001**
GGT (U/L)^b^	91.8 (52.0, 177.2)	85.0 (50.0, 148.0)	187.0 (67.5, 337.5)	**<0.001**
AFP (ng/mL)^b^	4.3 (2.9, 6.9)	4.2 (2.9, 6.8)	4.8 (3.0, 7.1)	0.580
Diagnosis, n (%)				**<0.001**
MASLD	720 (41%)	625 (41%)	95 (36%)	
HBV	157 (9%)	145 (10%)	12 (5%)	
HCV	201 (11%)	180 (12%)	21 (8%)	
Alcohol-associated	112 (6%)	75 (5%)	37 (14%)	
Other	175 (10%)	145 (10%)	30 (11%)	
Multiple	410 (23%)	342 (23%)	68 (26%)	
Non-selective beta-blocker use, n (%)	366 (27%)	300 (26%)	66 (36%)	0.002
Carvedilol use, n (%)	240 (14%)	198 (14%)	42 (16%)	0.348
Propanolol use, n (%)	128 (8%)	104 (7%)	24 (9%)	0.286
Endoscopic varices, n (%)	291 (37%)	206 (32%)	85 (59%)	**<0.001**

^a^ Bolded *p*-value ≤ 0.05 denotes statistical significance.

^b^ Values are represented in median (IQR).

Abbreviations: AFP, alpha-fetoprotein; ALP, alkaline phosphatase; ALT, alanine transaminase; AST, aspartate aminotransferase; BMI, body mass index; GGT, gamma-glutamyl transferase; HBV, hepatitis B virus; HCV, hepatitis C virus; INR, international normalized ratio; IQR, interquartile range; LSM, liver stiffness measurement; MASLD, metabolic dysfunction–associated steatotic liver disease; MELD, Model for End-Stage Liver Disease.

When stratified by the presence of at least one decompensation episode, patients who experienced liver decompensation had higher baseline LSM (27.7 vs. 21.8 kPa, *p*<0.001), MELD (10.0 vs. 8.0, *p*<0.001), lower platelet levels (107 vs. 159.5 ×10^9^/L, *p*<0.001), and lower albumin levels (38.0 vs. 42.0 g/L, *p*<0.001). Hypertension and obesity were less common among those who decompensated (45% vs. 60%, *p*<0.001; 35% vs. 50%, *p*<0.001), respectively. The distribution of etiology differed markedly, where ALD was over-represented among those who decompensated (14% vs. 5%), while HBV (5% vs. 10%) and HCV (8% vs. 12%) were under-represented. Endoscopic varices at baseline were more common in those who subsequently experienced decompensation (59% vs. 32%, *p*<0.001). A higher proportion of patients who decompensated were on NSBB use at baseline (36% vs. 26%, *p*=0.002), likely reflective of their more advanced baseline fibrosis stage. As these baseline comparisons are stratified by eventual decompensation status, they do not incorporate time-to-event and are intended only as descriptive, exploratory summaries. Transition-specific predictors of first decompensation are therefore reported using Cox models in Supplemental Table S2, https://links.lww.com/HC9/C482.

### Probability of first decompensation or death from baseline cACLD state

Following exclusion of patients with HCC development or receipt of liver transplantation during follow-up, 1585 patients were included in the main multistate analysis (Figure 1). Overall, 215 (13.6%) patients experienced first liver decompensation in the main analysis cohort. In the main multistate model adjusted for age and sex, progression to first decompensation from cACLD was infrequent during follow-up (Figure 2). From baseline, the transition rate to first decompensation was 3.10 per 100 person-years, with state occupancy probabilities of 0.04, 0.06, and 0.07 at 1, 3, and 5 years, respectively (Table 2). The cumulative incidence of first decompensation was 4.8% (95% CI: 3.7%–5.9%), 11.2% (95% CI: 9.5%–13.0%), and 16.4% (95% CI: 14.2%–18.6%) at 1, 3, and 5 years, respectively (Supplemental Table S3, https://links.lww.com/HC9/C482). Death from baseline occurred in 70 patients, at a rate of 1.01 per 100 person-years, with corresponding state occupancy probabilities of 0.01, 0.03, and 0.06 at 1, 3, and 5 years, respectively. Overall, most patients remained in the baseline cACLD state throughout follow-up, with baseline state occupancy probabilities of 0.95, 0.88, 0.81, and 0.72 at 1, 3, 5, and 10 years, respectively (Supplemental Table S4, https://links.lww.com/HC9/C482). When stratified by etiology of liver disease, the patients with ALD had the highest rate of first decompensation from baseline as compared with MASLD, HBV, or HCV, occurring at 10.86 per 100 person-years. A similar trend was observed for the transition from baseline cACLD to death, occurring in ALD patients at 1.81 per 100 person-years, as compared with <1 per 100 person-years in the other etiologies (Table 3).

**FIGURE 1:**
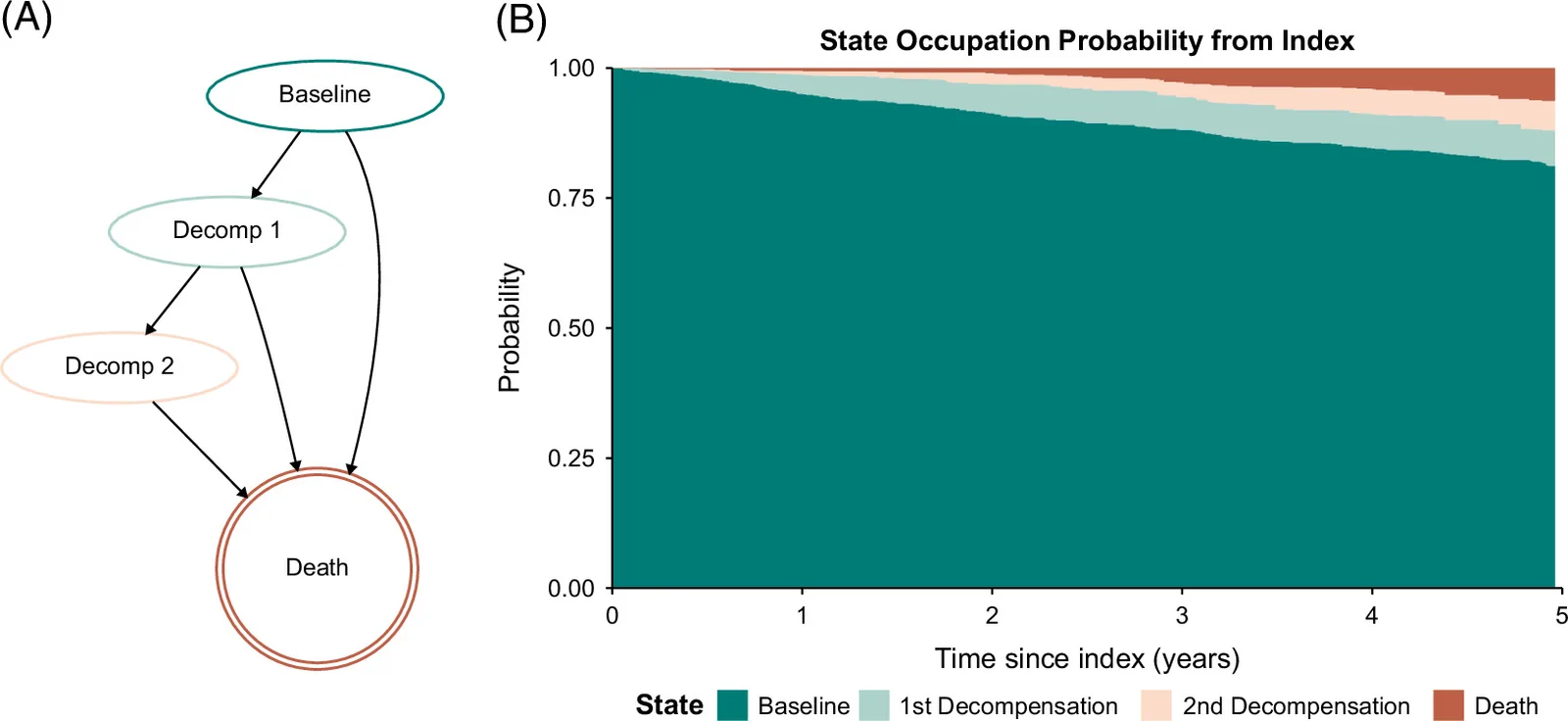
Multistate model structure and estimated state occupation probabilities. (A) Transitions from baseline to first decompensation, from first to second decompensation, and from each non-terminal state to death. (B) Estimated state occupation probabilities over 5 years from the index assessment (Baseline).

**FIGURE 2:**
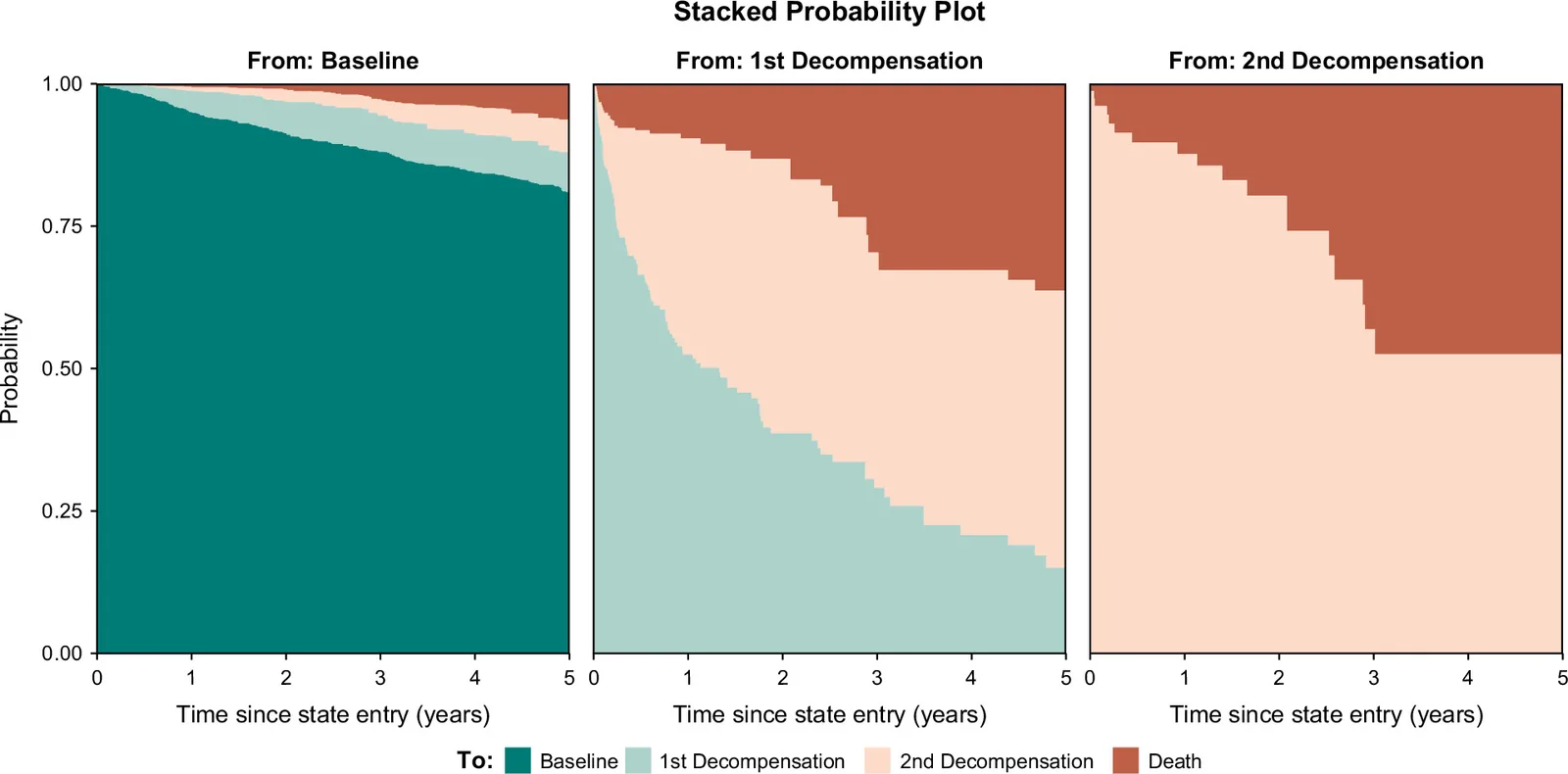
State occupation probabilities according to starting clinical state. Stacked probability plots showing the estimated probability of occupying each subsequent state over 5 years, from baseline, first decompensation, and second decompensation.

**TABLE 2:** Overall transition rates in the multistate model ^a^

Transition		Analysis		State occupancy probability		
		No. of events (n)	Event rate (per 100 person-years)	1 year	3 years	5 years
From baseline	1st decompensation	215	3.10	0.04 (0.03, 0.05)	0.06 (0.04, 0.08)	0.07 (0.04, 0.10)
	Death	70	1.01	0.01 (0.00, 0.01)	0.03 (0.01, 0.04)	0.06 (0.04, 0.09)
From 1st decompensation	2nd decompensation	92	42.73	0.38 (0.28, 0.47)	0.41 (0.26, 0.57)	0.49 (0.32, 0.65)
	Death	20	9.29	0.10 (0.04, 0.15)	0.30 (0.15, 0.44)	0.36 (0.21, 0.52)
From 2nd decompensation	Death	19	13.87	0.12 (0.03, 0.22)	0.43 (0.22, 0.64)	0.47 (0.26, 0.69)

^a^ Model adjusted for sex and age.

**TABLE 3:** Transition rates^a^ by etiology in the multistate model ^b^

Transition		Analysis		State occupancy probability		
		No. of events (n, %)	Event rate (per 100 person-years)	1 year	3 years	5 years
MASLD/MetALD						
From baseline	1st decompensation	85	2.83	0.03 (0.01, 0.04)	0.04 (0.01, 0.07)	0.07 (0.00, 0.13)
	Death	22	0.73	0.00 (0.00, 0.00)	0.02 (0.00, 0.04)	0.03 (0.00, 0.05)
From 1st decompensation	2nd decompensation	40	46.34	0.37 (0.20, 0.55)	0.40 (0.03, 0.78)	0.52 (0.14, 0.90)
	Death	4	4.63	0.05 (0.00, 0.13)	0.36 (0.00, 0.73)	0.36 (0.00, 0.73)
From 2nd decompensation	Death	8	17.57	0.21 (0.00, 0.44)	0.64 (0.21, 1.00)	0.64 (0.21, 1.00)
HBV						
From baseline	1st decompensation	12	1.39	0.01 (0.00, 0.04)	0.03 (0.00, 0.07)	0.00 (0.00, 0.04)
	Death	8	0.93	0.01 (0.00, 0.03)	0.04 (0.01, 0.07)	0.05 (0.01, 0.09)
From 1st decompensation	2nd decompensation	5	40.17	0.85 (0.58, 1.00)	0.00 (0.00, 0.00)	0.21 (0.00, 0.61)
	Death	0	0	0.00 (0.00, 0.00)	0.85 (0.58, 1.00)	0.85 (0.58, 1.00)
From 2nd decompensation	Death	1	26.07	0.00 (0.00, 0.00)	1.00 (1.00, 1.00)	1.00 (1.00, 1.00)
HCV						
From baseline	1st decompensation	13	1.46	0.02 (0.00, 0.05)	0.01 (0.00, 0.04)	0.00 (0.00, 0.10)
	Death	5	0.56	0.01 (0.00, 0.02)	0.02 (0.00, 0.04)	0.04 (0.00, 0.07)
From 1st decompensation	2nd decompensation	7	48.68	0.39 (0.05, 0.73)	0.58 (0.22, 0.94)	0.72 (0.15, 1.00)
	Death	2	13.91	0.34 (0.00, 0.71)	0.34 (0.00, 0.71)	0.34 (0.00, 0.71)
From 2nd decompensation	Death	0	0	0.00 (0.00, 0.00)	0.00 (0.00, 0.00)	0.00 (0.00, 0.00)
Alcohol-associated						
From baseline	1st decompensation	36	10.86	0.11 (0.04, 0.17)	0.21 (0.10, 0.33)	0.34 (0.20, 0.48)
	Death	6	1.81	0.00 (0.00, 0.00)	0.01 (0.00, 0.03)	0.04 (0.00, 0.09)
From 1st decompensation	2nd decompensation	15	47.62	0.46 (0.25, 0.66)	0.68 (0.49, 0.87)	0.55 (0.24, 0.87)
	Death	5	15.87	0.08 (0.00, 0.17)	0.08 (0.00, 0.17)	0.22 (0.00, 0.50)
From 2nd decompensation	Death	4	19.58	0.12 (0.00, 0.31)	0.12 (0.00, 0.31)	0.29 (0.00, 0.68)

^a^ Blank cells indicate estimates that were not computed due to insufficient observations.

^b^ Model adjusted for sex and age.

Abbreviations: HBV, hepatitis B virus; HCV, hepatitis C virus; MASLD, metabolic dysfunction–associated steatotic liver disease; MetALD, metabolic dysfunction– and alcohol-associated liver disease.

### Probability of second decompensation or death following the first decompensating event

In all, 2 in 5 (42.8%) of those patients who experienced a first decompensating event experienced a second decompensating event over a median further follow-up time of 0.42 years (IQR: 0.09–1.41) after the first decompensation. The rate of a second decompensation was higher following the first decompensation, at 42.73 per 100 person-years. The state occupancy probabilities were 0.38, 0.41, and 0.49 at 1, 3, and 5 years, respectively (Table 2). Among patients who experienced a first decompensation, the cumulative incidence of second decompensation was 39.5% (95% CI: 31.8%–47.1%), 61.2% (95% CI: 52.0%–70.4%), and 71.2% (95% CI: 61.6%–80.8%) at 1, 3, and 5 years after first decompensation, respectively. Compared with the baseline, death after first decompensation increased to 9.29 per 100 person-years. Corresponding death probabilities reached 0.10, 0.30, and 0.36 at 1, 3, and 5 years, respectively, after the first decompensating event (Table 2). When stratified by etiologies, patients with HBV had the lowest rate of second decompensation following the first decompensation, occurring at 40.17 per 100 person-years.

### Probability of death following the second decompensating event

After the second decompensation, mortality was high at 13.87 per 100 person-years, with probabilities of death of 0.12, 0.43, and 0.47 at 1, 3, and 5 years (Table 2). Collectively, these estimates underscore a low short-term event burden while compensated, a marked inflection after the first decompensation, and very poor medium-term survival following a second event (Figure 2). When stratified by etiologies, patients with HBV had the highest rate of death following the second decompensating event, occurring at 26.07 per 100 person-years (Table 3).

### Predictors of disease progression and mortality across multistate transitions

From the baseline cACLD state, absence of hypertension, absence of hyperlipidemia, non-Asian ethnicity, and non-obesity at baseline were associated with a higher hazard of a first decompensation event. Among laboratory markers, higher MELD, INR, bilirubin, AST, ALP, and GGT were associated with an increased risk. Conversely, a lower platelet count, serum creatinine, sodium, and albumin were associated with the first decompensation. Non-selective beta-blocker use was also associated with a higher risk of first decompensation, although this could be more reflective of baseline severity of liver disease.

From baseline cACLD to death, mortality was chiefly associated with increased age, MELD, serum creatinine, and ALP. Obesity was again inversely associated with mortality. BMI, serum sodium, albumin, ALT, AST, and AFP are also inversely associated with mortality. Following the first decompensation, higher serum creatinine was associated with second decompensation, and the absence of diabetes was associated with mortality. Other predictors of state transitions can be found in Supplemental Table S2, https://links.lww.com/HC9/C482.

### Sensitivity analysis incorporating HCC and liver transplant as absorbing states

In the sensitivity multistate model incorporating HCC and liver transplantation as additional transition states (Supplemental Figure S2, https://links.lww.com/HC9/C482), the overall pattern of disease progression remained largely consistent with the main analysis. From baseline cACLD, progression to first decompensation remained infrequent, with 265 patients experiencing a first decompensating event at a rate of 3.39 per 100 person-years (Supplemental Table S5, https://links.lww.com/HC9/C482). Corresponding state occupancy probabilities for first decompensation were 0.03, 0.05, and 0.07 at 1, 3, and 5 years, respectively. Death from baseline occurred at a lower rate of 0.91 per 100 person-years, with probabilities of 0.01, 0.02, and 0.05 at 1, 3, and 5 years (Supplemental Figure S3, https://links.lww.com/HC9/C482). HCC development from baseline occurred in 135 patients at a rate of 1.73 per 100 person-years, while transplantation was rare, occurring in only 6 patients at 0.08 per 100 person-years.

Following the first decompensation, the risk of further clinical deterioration remained substantially higher. Second decompensation occurred in 103 patients at a rate of 40.41 per 100 person-years, with state occupancy probabilities of 0.35, 0.35, and 0.42 at 1, 3, and 5 years, respectively. Death after first decompensation occurred at 7.45 per 100 person-years, with probabilities of 0.08 at 1 year to 0.30 at 5 years. After the second decompensation, mortality remained high, occurring at 12.77 per 100 person-years, with death probabilities of 0.11, 0.39, and 0.43 at 1, 3, and 5 years, respectively (Supplemental Figure S3 https://links.lww.com/HC9/C482). Overall, inclusion of HCC and transplantation did not materially alter the key findings of the main model: event burden was low while patients remained compensated, but the risk of subsequent decompensation and death increased markedly after the first decompensating event.

## DISCUSSION

This multicenter study included 1777 patients with cACLD from 18 centers, and we utilized a multistate model to provide a comprehensive description of the clinical course of cirrhosis. Our study demonstrates that most patients remained in the same compensated state, with <1 in 5 (19%) progressing to a decompensated state or death at 5 years. However, once a first decompensating event was reached, rates of subsequent clinical deterioration rose steeply thereafter, wherein over 80% of those patients would experience a second decompensation or death within 5 years. Following the second decompensating event, the 5-year prognosis is poor, with approximately only 1 in 2 patients alive at 5 years. Features such as non-obesity and MELD score identified patients at higher risk of decompensation and/or death while still in the compensated stage. When stratified by baseline liver disease etiology, we found that patients with ALD had the highest rate of progression to decompensated liver disease and death from baseline, and patients with HBV had the lowest rate of progression from first decompensation to subsequent decompensation.

Our study findings align with those in the literature, where a broad survival gap has been reported between compensated and decompensated cirrhosis, with an expected median survival of 12 years versus 2 years, respectively. Progression from compensated to decompensated cirrhosis occurs at 5%–7% annually.^2^^,^^16^ In our study, we report the cumulative incidence of first decompensation from baseline to be 4.8%, 11.2%, and 16.4% at 1, 3, and 5 years, respectively. The first decompensating event was most commonly ascites in previous studies,^17^^–^^19^ which is also reflected in our study. Balcar et al^20^ reported that, following a first onset of ascites, over a median follow-up of 48.5 months, about 56% developed a second decompensating event and 42% died.^20^ Similar findings were seen in a multicenter study by D’Amico et al,^9^ where the 5-year cumulative incidences of decompensation following the first decompensating event was 52%. Our study mirrors the existing literature, where the 5-year state occupancy probability of second decompensation from the first decompensating event was 49%. However, we report a 5-year state occupancy probability of death from first decompensation of 36%, marginally lower than that seen in the multicenter study by D’Amico et al.^9^ Moreover, the 5-year cumulative incidence of death after further decompensation was 55%, compared with 47% of patients who progressed from second decompensation to death at 5 years in our study. These findings could have been attributed to the proportions of etiologies of cirrhosis. As our study cohort is predominantly Asian, HBV was more strongly represented (9% as compared with only 3% in the former study), and ALD was less represented (6% compared with 44%).^9^ However, this associative gradient could be confounded by unmeasured antiviral exposure/virologic activity and era heterogeneity and should be interpreted cautiously.

Consistent with previous work, predictors previously linked to first decompensation, such as higher MELD and lower albumin, were also associated with progression in our multistate analysis.^21^^–^^24^ In our study, obesity, hyperlipidemia, and hypertension were inversely associated with first decompensation, consistent with the paradox observed in more advanced liver disease.^25^^–^^27^ Following the first decompensation event, further decompensation was associated with higher baseline creatinine, as seen in previous studies.^9^^,^^20^ The association between NSBB use and first decompensation is likely confounded by indication, where sicker patients with more severe portal hypertension were preferentially prescribed these agents. Following the second decompensation, the borderline significant inverse association with ALT with death is likely a reflection of the severity of liver disease, where liver enzymes fall as hepatocellular mass declines, rather than a protective effect.^28^^,^^29^

### Strengths and limitations

In this large multicenter cALCD cohort, we utilized a continuous-time multistate model, allowing estimation for transition-specific hazards and reporting of granular 1-, 3-, and 5-year state probabilities at crucial disease progression states in cirrhosis. By using a continuous-time multistate framework to model ordered transitions, we avoid overestimation when competing events exist.^11^ Our cohort’s predominantly Asian representation and etiology-stratified outputs provide regional and etiology-relevant data, which complement existing predominantly Western literature.^2^^,^^9^^,^^17^^,^^30^ However, as an observational study, there are inherent limitations. First, we lacked granular data on key disease-modifying factors, such as alcohol abstinence in alcohol-associated liver disease and longitudinal metabolic control or weight change in MASLD, which are known to significantly influence disease progression and may influence observed differences between etiological subgroups. Second, medication exposures, such as NSBB dosage and time to initiation, were likely heterogeneous and confounded by indication. Furthermore, several post-decompensation transitions within etiology-stratified analyses were based on small event counts, particularly in viral hepatitis subgroups, resulting in wide confidence intervals and occasional non-estimable parameters. These findings should therefore be interpreted as exploratory rather than definitive. In addition, patients were not restricted to newly diagnosed cases at the time of index VCTE, and some may have been under clinical follow-up before their first VCTE assessment. Therefore, index VCTE should be interpreted as the study entry point rather than the onset of cACLD. This may introduce left-truncation or prevalent cohort bias, as patients with longer-standing compensated disease who had not yet decompensated before VCTE may be selectively represented. Moreover, as the cohort consisted of patients who underwent VCTE based on clinician judgment rather than systematic screening, not all patients with possible cACLD would have been captured. This may introduce selection bias, as patients referred for VCTE may differ systematically from those who were not tested, and may limit generalizability to the broader cACLD population. Lastly, because transitions to HCC and liver transplant were sparse, they were not modeled as additional states in the main analysis. While this approach allowed for stable estimation of core disease transitions, exclusion of these events may bias estimates toward patients with uninterrupted natural progression and should be considered when interpreting transition probabilities.

## CONCLUSION

In conclusion, our multicenter study charts the natural history of cACLD using a transition-aware, continuous-time framework and shows that, over 5 years, most patients remain compensated; however, once a first decompensation occurs, the probability of death significantly increases. Etiology-stratified estimates highlight important heterogeneity, which serves as a basis for personalized prediction of liver disease trajectory. Moreover, the granular 1-, 3-, and 5-year probabilities move beyond the traditional “median survival” figures for compensated and decompensated liver disease, enabling clinicians to perform individualized counseling and timely transplant referral. Our study cohort, being predominantly Asian represented and from an era where NSBBs were not yet widely adopted in patients with clinically significant portal hypertension, provides an important regional baseline against which evolving practice can be compared.

## Supplementary Material
