# Supplementary material for: A multicenter multistate modeling on the natural history of liver decompensation: Longitudinal analysis of 1777 patients
Source: Hepatol Commun. 2026 Sep 28;10(10):e1042. doi: 10.1097/HC9.0000000000001042 (PMC13623273; doi:10.1097/HC9.0000000000001042)
Supplement: Supplementary file 1 [file hc9-10-e1042-s001.docx]

# Supplementary Table 1: Individuals contributed by each centre in final study cohort

| **Centre *** | **Proportion (n, %)** |
| --- | --- |
| AIG Hospitals | 163 (9.2%) |
| National University Hospital | 515 (29.0%) |
| Wenzhou University | 46 (2.6%) |
| University of Michigan | 37 (2.1%) |
| Khon Khaen University | 62 (3.5%) |
| Saga University | 55 (3.1%) |
| Chinese University of Hong Kong | 134 (7.5%) |
| University of Arizona | 347 (19.5%) |
| Changi General Hospital | 143 (8.0%) |
| Prince of Songkla University | 20 (1.1%) |
| Apollo Hospitals (Kolkata) | 31 (1.7%) |
| Royal Prince Alfred Hospital | 26 (1.5%) |
| Kurume University | 1 (0.1%) |
| University of Sydney | 66 (3.7%) |
| Yokohama City University | 4 (0.2%) |
| Kyoto Prefectural University | 39 (2.2%) |
| Shinshu University | 41 (2.3%) |
| Jilin University | 47 (2.6%) |

* Centres contributing no eligible participants after application of exclusion criteria are not presented.

# Supplementary Table 2: Predictors of transitions in the multistate model ^a^

|  | **Hazard Ratio (95% CI); p value** | | | | |
| --- | --- | --- | --- | --- | --- |
| **Characteristic** | **Baseline - 1^st^ Decompensation** | **Baseline - Death** | **1^st^ Decompensation - 2^nd^ Decompensation** | **1^st^ Decompensation - Death** | **2^nd^ Decompensation - Death** |
| **Age, years** | 1.00 (0.99, 1.01); 0.702 | 1.08 (1.05, 1.10); **<0.001** | 1.01 (0.99, 1.03); 0.202 | 0.99 (0.95, 1.03); 0.677 | 1.04 (0.99, 1.09); 0.127 |
| **BMI, kg/m^2^** | 0.98 (0.96, 1.01); 0.188 | 0.91 (0.86, 0.96); **<0.001** | 1.01 (0.97, 1.05); 0.590 | 0.91 (0.83, 1.00); 0.059 | 0.89 (0.78, 1.01); 0.066 |
| **Sex** |  |  |  |  |  |
| Female | 1.00 (reference) | 1.00 (reference) | 1.00 (reference) | 1.00 (reference) | 1.00 (reference) |
| Male | 0.86 (0.66, 1.13); 0.288 | 1.18 (0.73, 1.91); 0.509 | 0.96 (0.64, 1.44); 0.835 | 0.75 (0.30, 1.86); 0.538 | 1.25 (0.50, 3.15); 0.637 |
| **Ethnicity** |  |  |  |  |  |
| Other | 1.00 (reference) | 1.00 (reference) | 1.00 (reference) | 1.00 (reference) | 1.00 (reference) |
| Asian | 0.58 (0.34, 0.99); **0.046** | 0.96 (0.23, 3.96); 0.958 | 1.11 (0.40, 3.04); 0.846 | 1.26 (0.17, 9.53); 0.825 | ^†^ |
| **Diabetes** |  |  |  |  |  |
| Yes | 1.00 (reference) | 1.00 (reference) | 1.00 (reference) | 1.00 (reference) | 1.00 (reference) |
| No | 1.04 (0.79, 1.37); 0.787 | 1.20 (0.74, 1.94); 0.453 | 0.79 (0.51, 1.23); 0.301 | 3.11 (1.22, 7.92); **0.017** | 1.36 (0.54, 3.43); 0.519 |
| **Hypertension** |  |  |  |  |  |
| Yes | 1.00 (reference) | 1.00 (reference) | 1.00 (reference) | 1.00 (reference) | 1.00 (reference) |
| No | 2.18 (1.67, 2.86); **<0.001** | 0.84 (0.50, 1.41); 0.506 | 0.71 (0.47, 1.08); 0.108 | 1.98 (0.75, 5.23); 0.168 | 0.69 (0.28, 1.73); 0.431 |
| **Hyperlipidemia** |  |  |  |  |  |
| Yes | 1.00 (reference) | 1.00 (reference) | 1.00 (reference) | 1.00 (reference) | 1.00 (reference) |
| No | 2.17 (1.66, 2.85); **<0.001** | 0.69 (0.41, 1.17); 0.169 | 0.87 (0.58, 1.31); 0.501 | 1.37 (0.54, 3.48); 0.509 | 0.56 (0.22, 1.41); 0.220 |
| **Obesity** |  |  |  |  |  |
| Obese | 1.00 (reference) | 1.00 (reference) | 1.00 (reference) | 1.00 (reference) | 1.00 (reference) |
| Non-obese | 1.48 (1.11, 1.98); **0.007** | 2.69 (1.53, 4.74); **<0.001** | 1.15 (0.74, 1.78); 0.535 | 3.15 (0.91, 10.90); 0.070 | 3.24 (0.92, 11.34); 0.066 |
| **MELD score** | 1.10 (1.07, 1.13); **<0.001** | 1.11 (1.07, 1.16); **<0.001** | 1.01 (0.94, 1.09); 0.705 | 0.89 (0.75, 1.05); 0.167 | 0.98 (0.81, 1.19); 0.822 |
| **INR** | 1.18 (1.02, 1.36); **0.025** | 1.03 (0.63, 1.66); 0.914 | 1.74 (0.60, 5.06); 0.312 | 1.68 (0.28, 10.07); 0.568 | 6.60 (0.48, 91.67); 0.160 |
| **Platelet (x 10^9^/L) ^#^** | 0.65 (0.57, 0.75); **<0.001** | 0.87 (0.64, 1.18); 0.371 | 1.17 (0.88, 1.55); 0.278 | 0.66 (0.43, 1.00); 0.049 | 1.77 (0.68, 4.60); 0.238 |
| **Creatinine (umol/L) ^#^** | 0.58 (0.40, 0.86); **0.006** | 3.13 (2.36, 4.16); **<0.001** | 1.85 (1.04, 3.29); **0.036** | 0.89 (0.26, 3.10); 0.854 | 1.31 (0.44, 3.92); 0.628 |
| **Sodium (mmol/L)** | 0.93 (0.88, 0.98); **0.006** | 0.82 (0.77, 0.88); **<0.001** | 0.99 (0.92, 1.07); 0.751 | 0.97 (0.83, 1.12); 0.640 | 0.83 (0.71, 0.97); 0**.**018 |
| **Bilirubin (umol/dL) ^#^** | 1.82 (1.57, 2.12); **<0.001** | 0.96 (0.67, 1.39); 0.846 | 0.92 (0.70, 1.21); 0.551 | 0.67 (0.35, 1.30); 0.237 | 0.59 (0.29, 1.22); 0.155 |
| **Albumin (g/L)** | 0.90 (0.88, 0.92); **<0.001** | 0.89 (0.86, 0.92); **<0.001** | 0.99 (0.96, 1.03); 0.724 | 0.99 (0.91, 1.08); 0.797 | 0.99 (0.93, 1.05); 0.649 |
| **ALT (U/L) ^#^** | 0.88 (0.72, 1.06); 0.174 | 0.41 (0.29, 0.59); **<0.001** | 1.04 (0.78, 1.39); 0.794 | 1.23 (0.64, 2.39); 0.536 | 0.47 (0.22, 1.01); 0.053 |
| **AST (U/L) ^#^** | 1.49 (1.22, 1.82); **<0.001** | 0.60 (0.39, 0.91); **0.016** | 0.97 (0.72, 1.31); 0.832 | 1.60 (0.86, 2.96); 0.137 | 0.72 (0.30, 1.70); 0.454 |
| **ALP (U/L) ^#^** | 2.35 (1.88, 2.95); **<0.001** | 2.44 (1.65, 3.60); **<0.001** | 0.92 (0.61, 1.37); 0.671 | 2.04 (0.99, 4.21); 0.055 | 1.07 (0.49, 2.32); 0.870 |
| **GGT (U/L) ^#^** | 2.05 (1.68, 2.49); **<0.001** | 1.64 (0.92, 2.95); 0.096 | 0.96 (0.70, 1.31); 0.803 | 1.78 (1.00, 3.16); 0.050 | 1.32 (0.61, 2.88); 0.477 |
| **AFP (ng/mL) ^#^** | 1.04 (0.81, 1.33); 0.745 | 0.42 (0.24, 0.74); **0.003** | 0.92 (0.63, 1.34); 0.657 | 0.98 (0.42, 2.29); 0.969 | 0.80 (0.42, 1.53); 0.505 |
| **Non-selective beta blocker use** |  |  |  |  |  |
| Yes | 1.00 (reference) | 1.00 (reference) | 1.00 (reference) | 1.00 (reference) | 1.00 (reference) |
| No | 0.49 (0.35, 0.68); **<0.001** | 1.06 (0.52, 2.15); 0.872 | 0.78 (0.48, 1.28); 0.331 | 1.21 (0.11, 13.45); 0.875 | 0.51 (0.16, 1.61); 0.249 |
| **Carvedilol use** |  |  |  |  |  |
| Yes | 1.00 (reference) | 1.00 (reference) | 1.00 (reference) | 1.00 (reference) | 1.00 (reference) |
| No | 0.81 (0.57, 1.15); 0.242 | 1.16 (0.58, 2.35); 0.669 | 0.75 (0.44, 1.27); 0.288 | 3.46 (0.46, 26.00); 0.228 | 0.50 (0.16, 1.56); 0.233 |
| **Propanolol use** |  |  |  |  |  |
| Yes | 1.00 (reference) | 1.00 (reference) | 1.00 (reference) | 1.00 (reference) | 1.00 (reference) |
| No | 0.57 (0.36, 0.88); **0.012** | 3.59 (0.50, 25.88); 0.205 | 0.91 (0.50, 1.63); 0.746 | ^†^ | 0.84 (0.24, 2.90); 0.777 |
| **Endoscopic varices** |  |  |  |  |  |
| Yes | 1.00 (reference) | 1.00 (reference) | 1.00 (reference) | 1.00 (reference) | 1.00 (reference) |
| No | 0.33 (0.22, 0.48); **<0.001** | 2.60 (1.01, 6.69); **0.047** | 0.62 (0.36, 1.07); 0.089 | 0.21 (0.02, 1.79); 0.153 | 1.00 (0.36, 2.80); 0.999 |

**Legend:** 95% CI, 95% confidence interval; BMI, body mass index; MELD, model for end-stage liver disease; INR, international normalized ratio; MASLD, metabolic dysfunction-associated steatotic liver disease; HBV, hepatitis B virus; HCV, hepatitis C virus.

**^#^** Values are log-transformed.

* Bolded p-value ≤ 0.05 denotes statistical significance.

^†^ Model unstable due to sparse events; HR not interpretable.

^a^ Univariable models fitted for each predictor.

# Supplementary Table 3: Cumulative incidence of first and second decompensation ^a^

| **Group** | **Cumulative incidence (%)** | | | | | |
| --- | --- | --- | --- | --- | --- | --- |
|  | **First decompensation** | | | **Second decompensation ^b^** | | |
|  | **1-year** | **3-year** | **5-year** | **1-year** | **3-year** | **5-year** |
| Overall | 4.8 (3.7-5.9) | 11.2 (9.5-13.0) | 16.4 (14.2-18.6) | 39.5 (31.8-47.1) | 61.2 (52.0-70.4) | 71.2 (61.6-80.8) |
| MASLD/MetALD | 3.1 (1.7-4.4) | 8.2 (5.8-10.5) | 15.6 (12.2-19.0) | 41.3 (29.1-53.6) | 73.7 (57.4-90.0) | 79.6 (61.2-97.9) |
| HBV | 4.5 (1.0-8.1) | 6.2 (2.0-10.4) | 7.2 (2.6-11.7) | 55.0 (13.5-96.5) | 55.0 (13.5-96.5) | 77.5 (28.8-100.0) |
| HCV | 3.6 (0.8-6.5) | 4.9 (1.6-8.3) | 7.4 (3.1-11.8) | 42.7 (11.4-74.1) | 53.0 (20.2-85.8) | 68.4 (30.4-100.0) |
| Alcohol-associated | 13.3 (6.2-20.4) | 34.1 (23.0-45.1) | 46.2 (34.0-58.5) | 42.3 (22.2-62.4) | 62.3 (41.3-83.3) | 62.3 (41.3-83.3) |

**Legend:** 95% CI, 95% confidence interval.

^a^ Death before decompensation as competing risk.

^b^ Among individuals who experienced first decompensation.

# Supplementary Table 4: State occupancy probabilities in the multistate model ^a^

|  | | **State occupancy probabilities (95% CI)** | | | |
| --- | --- | --- | --- | --- | --- |
| **Transition** | | **1-year** | **3-year** | **5-year** | **10-year** |
| From baseline | Baseline | 0.95 (0.94, 0.96) | 0.88 (0.86, 0.90) | 0.81 (0.78, 0.84) | 0.72 (0.68, 0.76) |
|  | 1^st^ decompensation | 0.04 (0.03, 0.05) | 0.06 (0.04, 0.08) | 0.07 (0.04, 0.10) | 0.02 (0.00, 0.03) |
|  | 2^nd^ decompensation | 0.01 (0.00, 0.01) | 0.03 (0.01, 0.05) | 0.06 (0.03, 0.08) | 0.05 (0.01, 0.10) |
|  | Death | 0.01 (0.00, 0.01) | 0.03 (0.01, 0.04) | 0.06 (0.04, 0.09) | 0.21 (0.15, 0.26) |
| From 1^st^ decompensation | 1^st^ decompensation | 0.52 (0.43, 0.62) | 0.29 (0.18, 0.40) | 0.15 (0.06, 0.24) | 0.00 (0.00, 0.00) |
|  | 2^nd^ decompensation | 0.38 (0.28, 0.47) | 0.41 (0.26, 0.57) | 0.49 (0.32, 0.65) | 0.38 (0.12, 0.65) |
|  | Death | 0.10 (0.04, 0.15) | 0.30 (0.15, 0.44) | 0.36 (0.21, 0.52) | 0.62 (0.35, 0.89) |
| From 2^nd^ decompensation | 2^nd^ decompensation | 0.88 (0.78, 0.97) | 0.57 (0.36, 0.78) | 0.53 (0.31, 0.74) | 0.38 (0.10, 0.67) |
|  | Death | 0.12 (0.03, 0.22) | 0.43 (0.22, 0.64) | 0.47 (0.26, 0.69) | 0.62 (0.33, 0.90) |

**Legend:** 95% CI, 95% confidence interval

^a^ Model adjusted for sex and age.

# Supplementary Table 5: Overall transition rates in the multistate model including HCC and liver transplant as absorbing states ^a^

|  | | **Analysis** | | **State occupancy probability** | | |
| --- | --- | --- | --- | --- | --- | --- |
| **Transition** | | **No. of events (n, %)** | **Event rate (per 100 person-years)** | **1-year** | **3-year** | **5-year** |
| From baseline | 1^st^ decompensation | 265 | 3.39 | 0.03 (0.02, 0.04) | 0.05 (0.04, 0.07) | 0.07 (0.04, 0.10) |
|  | Death | 71 | 0.91 | 0.01 (0.00, 0.01) | 0.02 (0.01, 0.03) | 0.05 (0.03, 0.07) |
|  | HCC | 135 | 1.73 | 0.01 (0.00, 0.01) | 0.03 (0.02, 0.04) | 0.06 (0.04, 0.07) |
|  | Transplant | 6 | 0.08 | 0.00 (0.00, 0.00) | 0.00 (0.00, 0.00) | 0.00 (0.00, 0.01) |
| From 1^st^ decompensation | 2^nd^ decompensation | 103 | 40.41 | 0.35 (0.26, 0.43) | 0.35 (0.21, 0.48) | 0.42 (0.27, 0.56) |
|  | Death | 19 | 7.45 | 0.08 (0.03, 0.12) | 0.25 (0.12, 0.37) | 0.30 (0.16, 0.43) |
|  | HCC | 13 | 5.10 | 0.06 (0.02, 0.10) | 0.13 (0.05, 0.20) | 0.13 (0.05, 0.20) |
|  | Transplant | 27 | 10.59 | 0.03 (0.00, 0.07) | 0.03 (0.00, 0.07) | 0.03 (0.00, 0.07) |
| From 2^nd^ decompensation | Death | 19 | 12.77 | 0.11 (0.03, 0.20) | 0.39 (0.19, 0.59) | 0.43 (0.23, 0.64) |
|  | HCC | 6 | 4.03 | 0.01 (0.00, 0.04) | 0.07 (0.00, 0.14) | 0.07 (0.00, 0.14) |
|  | Transplant | 5 | 3.36 | 0.03 (0.00, 0.09) | 0.03 (0.00, 0.09) | 0.03 (0.00, 0.09) |

**Legend:** 95% CI, 95% confidence interval; IQR, interquartile range.

^a^ Model adjusted for sex and age.

# Supplementary Figure 1: Individuals stratified by etiology* of liver disease and time period of VCTE (before 2015 and 2015 and after)

Etiology by VCTE period

Number of Individuals

|  | Alcohol | HBV | HCV | MASLD/MetALD |
| --- | --- | --- | --- | --- |
| Before 2015 | 10 | 43 | 31 | 107 |
| 2015 and after | 102 | 114 | 170 | 613 |

# *Patients with other or multiple etiologies were not included in this chart.

# Supplementary Figure 2: State transition diagram for sensitivity analysis including HCC and liver transplant as absorbing states

# Supplementary Figure 3: Stacked probability plots for sensitivity analysis including HCC and liver transplantation as absorbing states
